# Incidental Gallbladder Duplication Detected on Imaging in an Adult With Nonspecific Gastrointestinal Symptoms: A Case Report

**DOI:** 10.7759/cureus.111638

**Published:** 2026-06-28

**Authors:** Maryana Radad, Omar Sharaiha

**Affiliations:** 1 Gastroenterology and Hepatology, Abdali Hospital, Amman, JOR

**Keywords:** biliary anomaly, computed tomography (ct ), gallbladder duplication, incidental radiological finding, ultrasound imaging

## Abstract

Gallbladder duplication is a rare congenital biliary anomaly that can present significant diagnostic and surgical challenges if unrecognized. We present the case of a 29-year-old male with nonspecific gastrointestinal symptoms in whom a gallbladder duplication was initially identified on ultrasound and further evaluated using computed tomography (CT), without evidence of inflammation or biliary obstruction. The anomaly was considered incidental and unrelated to the presenting symptoms, and no surgical intervention was required. Accurate preoperative identification of this variant is vital to prevent iatrogenic complications should the patient require a cholecystectomy in the future.

## Introduction

Gallbladder duplication is a rare congenital anomaly of the biliary system, with an estimated incidence of approximately 1 in 4,000 births [1]. It may present as either a bilobed gallbladder or true duplication, with variable cystic duct anatomy as described in the Harlaftis classification [2].

In most cases, this anomaly is detected incidentally during imaging or surgery. Ultrasonography is typically the first imaging modality used in the evaluation of gallbladder duplication [3]. Although many patients remain asymptomatic, recognition of this anatomical variant is clinically important. Unrecognized duplication has been associated with complications such as incomplete cholecystectomy and bile duct injury [1,4].

From an embryological perspective, gallbladder duplication is thought to result from abnormal division of the cystic bud during development [5]. Computed tomography (CT) and magnetic resonance cholangiopancreatography (MRCP) have been reported as useful adjunctive modalities for further characterization of gallbladder duplication and associated biliary anatomy [6]. Given its potential implications during future hepatobiliary procedures, accurate identification and reporting of this anomaly are important.

## Case presentation

A 29-year-old man presented with early satiety, abdominal fullness, fatigue, and reported unintentional 14 kg weight loss over one month. He denied abdominal pain, nausea, vomiting, jaundice, or changes in bowel habits. His family history was notable for colon cancer. 

Given the patient's significant weight loss and family history of colorectal cancer, upper endoscopy and colonoscopy were performed as part of the diagnostic evaluation. Upper endoscopy did not reveal significant pathology. Colonoscopy demonstrated melanosis coli consistent with chronic laxative use and did not reveal additional significant endoscopic abnormalities. Biopsies were not obtained, as there were no suspicious lesions requiring histopathological evaluation. Laboratory investigations, including complete blood count, liver function tests, inflammatory markers, and malignancy screening studies, were unremarkable. Cross-sectional abdominal imaging did not identify any masses, lymphadenopathy, or other abnormalities that could account for the patient's symptoms or weight loss.

Right upper quadrant ultrasonography demonstrated two adjacent, thin-walled anechoic structures within the gallbladder fossa, without wall thickening, pericholecystic fluid, or intraluminal calculi. A transverse view highlights the presence of two adjacent cystic structures (Figure 1), while a longitudinal view confirms two distinct gallbladder sacs (Figure 2). Contrast-enhanced CT confirmed the presence of two distinct fluid-density gallbladder sacs located adjacent within the gallbladder fossa (Figure 3).

**Figure 1:**
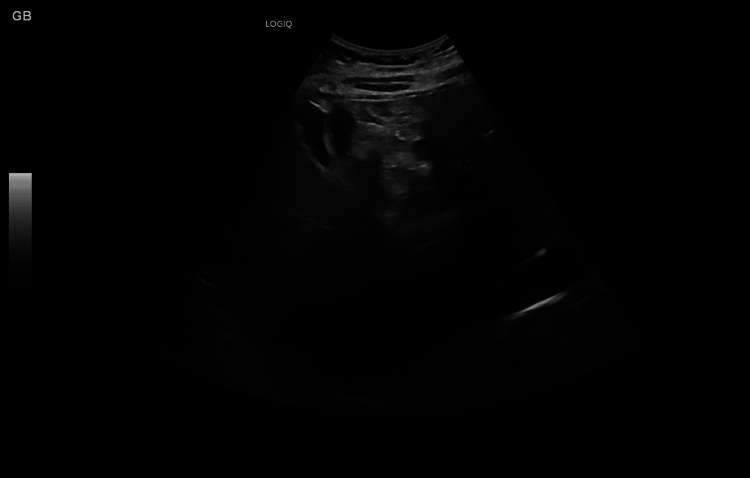
Transverse right upper quadrant ultrasound image demonstrating two adjacent thin-walled anechoic structures within the gallbladder fossa, consistent with gallbladder duplication.

**Figure 2:**
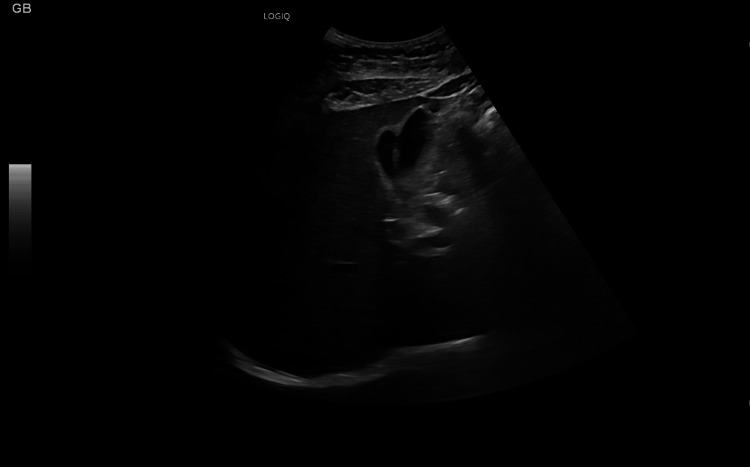
Longitudinal ultrasound view showing two distinct gallbladder sacs without wall thickening, pericholecystic fluid, or intraluminal calculi.

**Figure 3:**
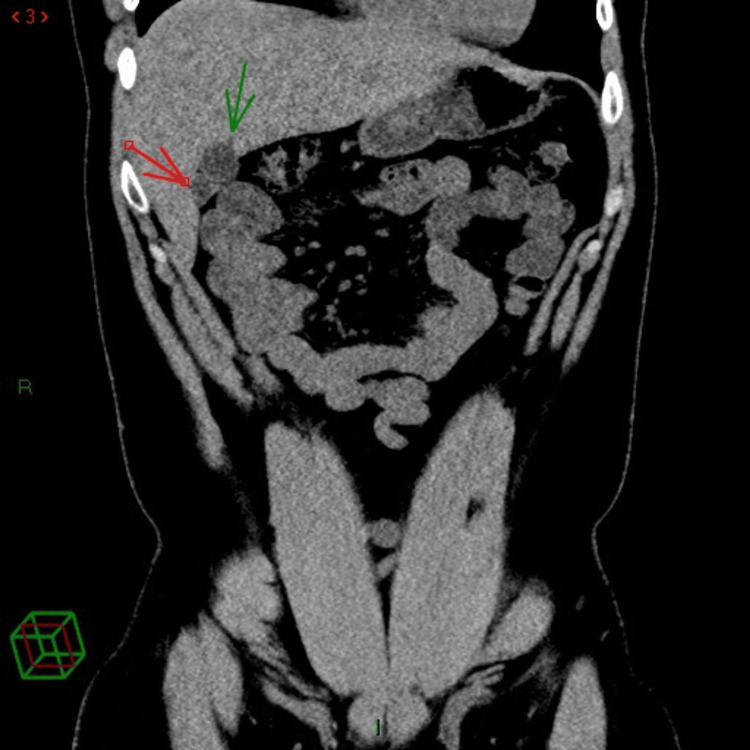
Coronal contrast-enhanced CT image demonstrating two adjacent gallbladder sacs within the gallbladder fossa. No biliary obstruction, masses, or lymphadenopathy are identified. The red arrow indicates one gallbladder, and the green arrow indicates the second gallbladder. CT, computed tomography.

No additional biliary imaging was deemed necessary, as there was no evidence of biliary obstruction, inflammation, or gallstone disease. Management focused on addressing the patient's gastrointestinal symptoms and chronic laxative use. The patient was reviewed after two weeks, with subsequent improvement in symptoms. Follow-up was continued as clinically indicated.

## Discussion

Gallbladder duplication is most often identified incidentally during imaging or surgery. Although frequently asymptomatic, recognition of this anatomical variant is important because failure to identify it preoperatively may increase the risk of operative complications during future hepatobiliary procedures [1,4,6]. Additional imaging modalities, such as CT and MRCP, can further characterize biliary anatomy and help exclude associated pathology. MRCP can provide detailed delineation of biliary anatomy and facilitate classification of gallbladder duplication, particularly when ultrasonography or CT findings are inconclusive [3,6].

Earlier classification systems, including those proposed by Boyden and Gross, described gallbladder duplication primarily according to anatomical morphology. However, the Harlaftis classification remains the most widely accepted system because it incorporates embryologic development and cystic duct anatomy, making it particularly useful for clinical and surgical planning [2].

In a bilobed gallbladder (vesica fellea divisa), two chambers share a common neck and cystic duct. In contrast, true gallbladder duplication (vesica fellea duplex) consists of two distinct gallbladders with separate cystic duct anatomy. The imaging findings in this case favored duplication; however, definitive classification was limited by the inability to fully delineate the cystic ducts on CT.

In this case, ultrasonography and CT imaging were sufficient to identify gallbladder duplication and exclude significant biliary pathology. However, cystic duct anatomy could not be definitively characterized, precluding precise classification. Given the absence of imaging evidence of cholecystitis or biliary obstruction, the duplication was considered unrelated to the presenting symptoms, which were more likely attributable to the patient's history of chronic laxative use and melanosis coli identified on colonoscopy.

Clinician familiarity with this anatomical variant assists in accurate radiologic reporting and helps surgeons mitigate the risk of intraoperative complications [1,6]. This case highlights the importance of thorough imaging evaluation in identifying rare biliary anatomical variants and facilitating safe future hepatobiliary surgical planning.

## Conclusions

Gallbladder duplication is an uncommon biliary anomaly that is often discovered incidentally during imaging performed for unrelated indications. This case highlights the importance of recognizing such variants and documenting them appropriately, as they may influence future hepatobiliary surgical planning. Careful imaging evaluation remains essential for accurate diagnosis and avoidance of potential operative complications.
